# Professionalism and Occupational Well-Being: Similarities and Differences Among Latin American Health Professionals

**DOI:** 10.3389/fpsyg.2017.00063

**Published:** 2017-01-25

**Authors:** Montserrat San-Martín, Roberto Delgado-Bolton, Luis Vivanco

**Affiliations:** ^1^Faculty of Social Sciences of Melilla, University of GranadaGranada, Spain; ^2^Education Committee Board, Hospital San Pedro of LogroñoLogroño, Spain; ^3^Center for Biomedical Research of La RiojaLogroño, Spain; ^4^National Centre of Documentation on BioethicsLogroño, Spain

**Keywords:** empathy, collaboration, lifelong learning, somatization, exhaustion, alienation, healthcare professionals, Latin America

## Abstract

**Context:** Empathy, teamwork, and lifelong learning are described as key elements of professionalism. The first recipients of their benefits are professionals themselves. Paradoxically, scarce studies have reported association between professionalism and occupational well-being. The main purpose of this study was to characterize the influence that empathy, teamwork, and lifelong learning, play in the occupational well-being of physicians and nurses working in Latin American healthcare institutions.

**Materials and Methods:** The Jefferson Scale of Empathy, the Jefferson Scale of Attitudes toward Physician-Nurse Collaboration, the Jefferson Scale of Physicians Lifelong Learning, and the Scale of Collateral Effects (somatization, exhaustion, and work alienation), were administered to 522 physicians and nurses working in institutions of Mexico, Colombia, Ecuador, and Argentina. Internal reliability was calculated. Gender and discipline were used as explanatory variables in comparison analysis. Two-way analysis of variance was performed to examine differences due to the main effects of the gender, and discipline, and to determine possible combined effects. Correlation analysis was performed to measure associations between collateral effects and age, and between collateral effects and professionalism.

**Results:** A total of 353 (68%) surveys were returned fully completed. Adequate reliability was confirmed in all instruments. No differences were found among countries for collateral effects. Correlation analysis confirmed in physicians an inverse association between empathy and collateral effects (*P* = -0.16; *p* < 0.05), and between collateral effects and lifelong learning (*P* = -0.18; *p* < 0.01). In nurses, this association was confirmed only for empathy (*P* = -0.19; *p* < 0.05). Important differences in the development of professionalism and in its effects on occupational well-being appeared associated to inter-professional collaboration and work roles. An inverse correlation between age and collateral effects was confirmed in physicians (*P* = -0.22; *p* < 0.001) and in nurses (*P* = -28; *p* < 0.001). Comparison by gender confirmed higher somatization in women physicians and nurses than in men groups (*p* < 0.001). On the other hand, comparison by discipline confirmed higher exhaustion and alienation in physicians than in nurses (*p* < 0.01).

**Conclusion:** The findings support the importance that empathy, teamwork, and lifelong learning have in practitioners’ health and welfare, and the role that cultural behaviors, associated to work professional models and social stereotypes, play in the interaction between professionalism and occupational well-being.

## Introduction

According to the Medical Professionalism Project (2002), an international consortium created by three leading medical organizations: the American Board of Internal Medicine (ABIM), the American College of Physicians and American Society of Internal Medicine (ACP-ASIM), and the European Federation of Internal Medicine, professionalism refers to the set of skills, values, and behaviors that characterize the essence of humanism in professional work. In physicians and nurses, this professionalism arises as an articulated body made up of professional traits and skills that constitute their professional work, regardless of the geographical, social, or cultural settings where it is carried out (Vivanco and Delgado-Bolton, 2015). The Royal College of Physicians (2005), remarks that recipients of the benefits of this professionalism are not only patients, but also healthcare professionals and, ultimately, the society as a whole. Efforts to foster professionalism in healthcare settings emphasize the qualities and attainments of physicians and nurses, beyond their required medical knowledge and clinical skills. Some educational organizations, as the Accreditation Council on Graduate Medical Education (ACGME), the Liaison Committee on Medical Education (LCME), or the Association of American Medical Colleges (AAMC), call attention to this critical educational objective (Cohen, 2006). Unfortunately, professionalism remains among the most difficult domains of doctor competence to assess (Veloski and Hojat, 2006). Although many promising approaches are under evaluation, no single measure or set of measurements has yet proven sufficiently reliable and valid to meet demanding psychometric criteria. In this scenario, Veloski and Hojat (2006) suggested to create a multi-score profile based on recognized elements of professionalism that can be measured. A multi-score measure, according to those authors, has two important advantages in comparison to a global measure: it can provide more complete information from characterized and independent domains, and it can identify specific elements that may need attention for medical educators and health managers. In the challenging work to define which elements of professionalism are capable to be psychometrically measured, Veloski and Hojat proposed three elements that are recognized components of professionalism: empathy, teamwork, and lifelong learning.

Empathy is described as a central attribute of the *humanistic* healthcare professionals (Arnold, 2002) that is also embedded in the three fundamental principles of professionalism described in the “Physicians’ charter” (2002): primacy of patient welfare, patient autonomy, and social justice. In healthcare settings, empathy is defined as a “predominantly *cognitive* (rather than an affective or emotional) attribute that involves an *understanding* (rather than feeling) of experiences, concerns and perspectives of the patient, combined with a capacity to *communicate* this understanding, and an *intention to help*” (Hojat, 2016, p. 74). In clinical encounters, empathy – as it was remarked at the beginning of this definition – also prevents to the negative effects caused by an intensive emotional involvement (Hojat, 2016). A large volume of literature supports the important role that empathy plays in patients’ adherence to treatment regimens, in their satisfaction with the healthcare provider and the healthcare system, and how this ability helps with coping with the disease (Hojat et al., 2015a). But not only patients, also healthcare professionals may perceive the benefits of being empathetic. According to Hojat (2016), physicians often perceive empathic relations with patients as meaningful interpersonal connections, and these relationships can serve as a buffer against dissatisfaction with the healthcare system and professional burnout. It has been demonstrated in physicians that empathic relationships with their patients provide an intrinsically joyful reward that serves as a remedy for the stress associated to their profession (Zuger, 2004). Empathy has been also identified as a protective factor against the stress experienced by clinicians (Shamasundar, 1999), as a potential factor for their well being (Hyyppä et al., 1991), and a protective factor against burnout in physicians (Thirioux et al., 2016), in physicians-in-training (Park et al., 2016), in nurses (Yu et al., 2016), and in medical students (Hojat et al., 2015b).

Teamwork and inter-professional collaboration work between physicians and nurses are behavioral examples of *respect* and *accountability* to others on the healthcare team. These two characteristics of teamwork are directly related with other two professional commitments of professionalism described in “Physicians’ charter”: the commitment to improving quality of care, and the commitment to professional responsibilities (Medical Professionalism Project, 2002). In clinical settings, this teamwork refers to a set of abilities that nurses and physicians have when they are able to work together cooperatively, sharing responsibilities for solving problems and making decisions to formulate and carry out plans centered on patients’ care (Hojat et al., 1999). In this frame, teamwork can be described as a “complementary” work model where the emphasis is stressed on interdisciplinary education, communication skills, shared autonomy, and mutual authority (Vivanco and Delgado-Bolton, 2015). This model of inter-professional relationship is not a recent phenomenon and has been the subject of several World Health Organization reports (Hammick et al., 2007; Thistlethwaite, 2012). By definition, this model is opposite to a “hierarchical” one where medicine is placed above nursing in patient-care responsibilities while nurses are viewed as “handmaidens” of physicians (Tang et al., 2013). In societies where a “hierarchical” model is dominant, nurses have little autonomy while most of patient-care decisions are carried out by physicians. In consequence, the risk of teamwork and communication failures increases. Empirical research has shown that those failures are the leading causes of worldwide patient safety incidents in health care institutions (Abdi et al., 2015; Hailu et al., 2016). Furthermore, working in hierarchical work environments also increases the risk of burnout in nurses (Hakanen et al., 2014). On the other hand, it has been demonstrated that the benefits that inter-professional collaborative work has in improving moral distress of caregivers and the quality of patients’ care (Piers et al., 2014; Lancaster et al., 2015).

Finally, lifelong learning is described as a third key element of professionalism. According to Veloski and Hojat (2006), lifelong learning is a component of both *excellence* and *self-regulatory and accountable* behavior to ensure quality of care. The “Physicians’ charter” explicitly put lifelong learning as a central component of the commitment of professional competence, and as a necessarily requirement to uphold scientific standards, to promote research, to create new knowledge and to ensure its appropriate use in patients care (Medical Professionalism Project, 2002). This attribute involves an asset of self-initiated activities (behavioral aspect) and information-seeking skills (capabilities) that are activated in individuals with a sustained motivation (predisposition) to learn and the ability to recognize their own learning needs (Hojat et al., 2009). There is a demonstrated association between lifelong learning and some indicators of occupational well being such as motivation, professional accomplishments, career satisfaction, and professional commitment (Hojat et al., 2009). This association is also in accordance with the Job Demand-Control model (JDC model) on learning and work characteristics (Karasek, 1979). According to the JDC model, having abilities to control work activities (skill discretion and decision authority) reduces workers’ stress but increases their attitudes toward learning. In a recent study, differences reported on lifelong learning in Ugandan nurses bring new elements for a better understanding of the complex interaction that takes place between this element and job demands, job control, and social support (Muliira et al., 2012). Social support is defined as the overall level of helpful social interactions available in the job from coworkers and supervisors that make workers feel valued and enmeshed in a network of communication and mutual obligation (Johnson and Hall, 1988; Karasek and Theorell, 1990). Social support plays an essential role in work-health interaction and learning’s improvement. For example, it is demonstrated that nurses’ attitudes toward lifelong learning are enhanced in workplaces with high job control and high supervision support (Doorn et al., 2016). In contrast, work overload and poor or null supervision act as barriers to foster lifelong learning (Muliira et al., 2012).

From a cross-cultural approach, differences between Anglo-Saxon America and Latin or Hispanic America have been pointed out by sociologists and economists (Belaunde, 1923; Saltalamacchia, 2014). Despite important social, political, and economical differences among Latin American countries, there still persists an idea of a common cultural identity. In fact, there are no big differences among Latin American countries in terms of medical and nursing educational curricula (Andrade, 1978) and in public health policies (Atun et al., 2015). This is reflected in findings reported in Latin American physicians in relation to medical empathy (Alcorta-Garza et al., 2016), or attitudes toward inter-professional collaboration and lifelong learning (San Martín et al., 2016). In relation to professional roles and inter-professional relationships, two studies reported a “hierarchical” model as dominant in Mexican institutions (Hojat et al., 2001, 2003). Despite this unique evidence reported is only from Mexican institutions, it is reasonable that a similar tendency may appear in institutions from other Latin American countries since medical education systems and social stereotypes associated to medicine and nursing are similar. In fact, findings reported in two recent studies on well being and work distress in Latin American institutions may offer new evidence in support of a common cultural perception of the physicians’ professional role (Grau et al., 2009; Ochoa and Blanch, 2016). In the first study (Grau et al., 2009), with Latin American healthcare professionals from Argentina, Uruguay, Mexico, Ecuador, Peru, Colombia, Guatemala, and El Salvador, differences in global perception of burnout were reported when the Argentinean group was compared to the other Latin American countries, but also when physicians were compared to the other healthcare professionals. In the second study (Ochoa and Blanch, 2016), performed only with physicians from Colombia, Brazil, Chile, and Venezuela, no differences were reported by country neither for somatization, exhaustion, or work alienation. Based on these findings, it is possible that not only differences in working conditions among countries, but also the dominance of a “hierarchical” model associated to the discipline may influence the presence of differences of workload among healthcare professionals of Latin America.

Despite the scarce number of studies exploring the relation among cultural and social factors, occupational well-being, and professionalism in Latin American institutions, the above mentioned findings offer an important preliminary support. Taking this into consideration, this study was designed with the main purpose of determining the type of influence that some elements of professionalism (empathy, teamwork, and lifelong learning) play in the occupational health and well-being of physicians and nurses who are working in healthcare institutions of four Latin American countries. With this purpose, and based on previous reports on other locations mentioned above, three research hypotheses were tested: (i) Occupational health, measured by three indicators (somatization, exhaustion, and alienation), is not different when groups by country are compared; (ii) Professionalism’s measures (empathy, teamwork, and lifelong learning) vary according to discipline; and (iii) Despite these differences, professionalism plays a protective role against work distress for Latin American healthcare professionals.

## Materials and Methods

### Participants

The study was based on a sample of healthcare professionals (physicians and nurses) involved in direct patient care and who were working in four public healthcare institutions (general hospitals) with similar characteristics located in the provinces of Yucatán (Mexico), Bogota (Colombia), Santa Elena (Ecuador), and Río Negro (Argentina). Inclusion criteria were: healthcare professionals (physicians and nurses) who had completed their professional training and, at the time of the study, had a work contract directly with the referred institution. Exclusion criteria were: medical students and students of nursing, physicians-in-training, nursing assistants, or being healthcare professionals without a direct work contract with the institutions (working for tertiary parties). Healthcare professionals who complied with the inclusion criteria were invited to participate voluntarily and anonymously.

### Principal Measures

The participants completed the Scale of Collateral Effects (SCE) of the Questionnaire of General Labor Well-being. The SCE is a psychometrically sound instrument composed by the sub-scale of somatization, the sub-scale of exhaustion, and the sub-scale of alienation, that measures somatization, exhaustion, and work alienation, respectively (Blanch et al., 2010). The sub-scale of somatization includes 5 items, the sub-scale of exhaustion includes 4 items, and the sub-scale of alienation includes four items. All items are answered on a 7-point Likert-type scale (1 = never, 7 = always). Possible scores of the SCE range from 13 to 91 and the higher the score, the greater the side-effects self-perception.

To measure empathy the healthcare professional version of the Jefferson Scale of Empathy (JSE) was used. The JSE includes 20 items that measure empathetic behavior of physicians and health professionals in the context of patients’ care (Hojat et al., 2002; Alcorta-Garza et al., 2016). The JSE is answered in a 7-point Likert scale (1 = strongly disagree, 7 = strongly agree). A higher score in this scale is associated with a more empathetic behavior in the context of patients’ care.

To measure teamwork the Jefferson Scale of Attitudes toward Physician-Nurse Collaboration (JSAPNC) was used. The JSAPNC is a 15-item scale that measures attitudes toward physician-nurse collaborative relationships (Hojat et al., 1999). The JSAPNC is answered in a 4-point Likert scale (1 = strongly disagree, 4 = strongly agree). A higher score on this scale is associated with a more positive attitude toward physician-nurse collaborative relationships.

To measure lifelong learning the Jefferson Scale of Physicians Lifelong Learning (JeffSPLL), and its variant for other health professionals different than medicine was used. The JeffSPLL includes 14 items that measure the orientation toward lifelong learning of physicians (Hojat et al., 2009) and other health practitioners (Muliira et al., 2012). The JeffSPLL is answered in a 4-point Likert scale (1 = strongly disagree, 4 = strongly agree).

### Complementary Information

Information about age, gender, and discipline, was collected through a complementary survey.

### Procedures

Between 2014 and 2015, questionnaires including the instruments and the complementary surveys were administered to physicians and nurses of the participant institutions. The questionnaires consisted of paper forms provided together with an information letter in enclosed envelopes that were returned to the local researchers following a general protocol previously approved by an independent ethics committee (Ref. CEICLAR PI 199). All participant institutions provided administrative support to the process of distribution and collection of the questionnaires. The work was carried out in accordance with the Ethical principles for medical research involving human subjects of the Declaration of Helsinki, adopted by the 18th World Medical Association (WMA) and amended by the 64th WMA General Assembly in 2013. There was no potential risk for participants, and anonymity was guaranteed throughout the process.

### Statistical Assessment

Internal consistency reliability was calculated using Cronbach’s alpha coefficient. Following the guidelines suggested by the American Educational Research Association, values higher than 0.7 were considered satisfactory.

An analysis of variance was performed to determine differences in the punctuations of the SCE and its three sub-scales among the groups from Mexico, Colombia, Ecuador, and Argentina.

The scores of the SCE and its three sub-scales were considered as dependent variables, gender (men and women), and discipline (physicians and nurses) were used as explanatory variables. A two-way analysis of variance (2 × 2 design) was performed to examine the group differences due to the main effects of the gender (men versus women), and discipline (physicians versus nurses). A two-way interaction effects were analyzed to determine if there were differences on scores in subgroups defined by a combination of “gender by discipline”. For comparisons among all punctuations, global scores were transformed into a common adjusted score from 0 to 100.

Age, empathy, and attitudes toward inter-professional collaboration and lifelong learning were analyzed using a correlation analysis. In order to determine the most suitable correlation coefficient, normality was previously analyzed in each case.

All analyses were performed using R statistical software, version 3.1.1 for Windows. The statistical analyses of the data also included multilevel (Bliese, 2013), and nortest (Gross, 2012) packages.

## Results

Of the 522 healthcare professionals who received the questionnaires, 374 returned their surveys. From them, 353 were fully completed, giving an overall effective response rate of 68%. With regard to representativeness, this response rate was considerably higher than the typical response rate of 61% reported for mailed surveys to practitioners (Cummings et al., 2001), and similar than the mean rate of 68% reported in previous studies using mailed surveys to American practitioners (Cull et al., 2005). However, a response rate of at least 75% should be achieved to ensure representativeness of the sample for mailed surveys to professionals (Gough and Hall, 1977). Following a methodology proposed by other authors with a similar situation (Hojat et al., 2002), a comparison between the respondents and non-respondents on their disciplines (the only variable available for non-respondents) was performed to ensure that the respondents were representative in that regard. No significant differences in disciplines were observed between the respondents and the non-respondents.

The mean age was 38 years old with a 23 to 66 year-old age range (*SD* = 9.51). Sixty nine (18%) of the surveys returned were from Mexico; 85 (23%) from Colombia; 70 (19%) from Argentina; and 150 (40%) from Ecuador. The entire sample was composed by 225 (64%) physicians (107 men and 118 women physicians), and 128 (36%) nurses (36 men and 92 women nurses). The score distribution, descriptive statistics, and reliability for the four instruments used in this study are described in **Table 1**.

**Table 1:** Descriptive statistics and psychometric reliability of scales of collateral effects, empathy, teamwork, and lifelong learning in 353 Latin American physicians and nurses.

Statistics	SCE				JSE-HP	JSAPNC	JeffSPLL
				
	Global	SS1	SS2	SS3			
Possible range	13–91	5–35	4–28	4–28	20–140	15–60	14–56
Actual range	13–91	5–35	4–28	4–28	60–140	18–60	18–56
Mean	41	17	15	9	110	50	48
Standard deviation	18	8	7	6	15	7	6
Percentile							
25th	25	10	8	4	98	46	45
50th (Median)	41	16	15	8	111	51	49
75th	54	23	21	13	121	55	52
Reliability	0.92	0.85	0.91	0.83	0.79	0.81	0.83


*SCE, Scale of Collateral Effects; SS1, Sub-scale of somatization; SS2, Sub-scale of exhaustion; SS3, Sub-scale of alienation; JSE-HP, Jefferson Scale of Empathy HP-Version; JSAPNC, Jefferson Scale of attitudes toward physician-nurse collaboration; JeffSPLL, Jefferson Scale of physician lifelong learning.*

With regard to the first research hypothesis related to work health and country of residence, no differences were found among countries when the global score of the SCE was compared [*F*_(1,351)_ = 0.04; *p* = 0.85]. Similar situation was observed in scores of the sub-scales of somatization [*F*_(1,351)_ = 2.26; *p* = 0.13], exhaustion [*F*_(1,351)_ = 0.2; *p* = 0.65], and alienation [*F*_(1,351)_ = 0.98; *p* = 0.32]. Based on these preliminary findings, the entire sample was treated as a unique group in subsequent analyses for the three collateral effects assessed.

**FIGURE 1:**
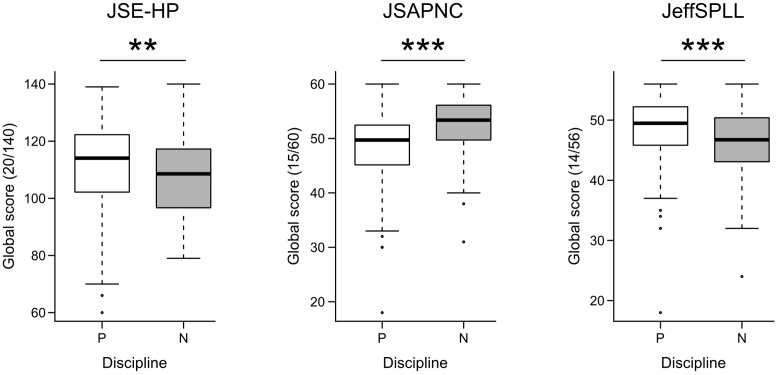
Comparisons by discipline, physicians (P) and nurses (N), in global scores of Jefferson Scale of Empathy (JSE-HP), Jefferson Scale of attitudes toward physician-nurse collaboration (JSAPNC), and Jefferson Scale of physicians’ lifelong learning (JeffSPLL); ^∗∗^*p <* 0.01; ^∗∗∗^*p <* 0.001.

Regarding the second research hypothesis related to professionalism and discipline, a *t*-test was performed to compare the punctuations for the three elements measured of professionalism. Comparison analysis showed that physicians’ group expressed higher empathetic orientation than nurses’ group (*t* = 2.61, *p* = 0.009). Physicians also showed higher abilities toward lifelong learning than nurses (*t* = 3.92, *p <* 0.001). On the other hand, nurses showed more positive attitudes toward physician-nurse collaboration than physicians (*t* = –6.68, *p* < 0.001). Those differences are shown in **Figure 1**.

Concerning the third research hypothesis related to professionalism as a protective factor against work distress in healthcare professionals, findings of the three collateral effects measured are summarized as follows:

### Somatization

**FIGURE 2:**
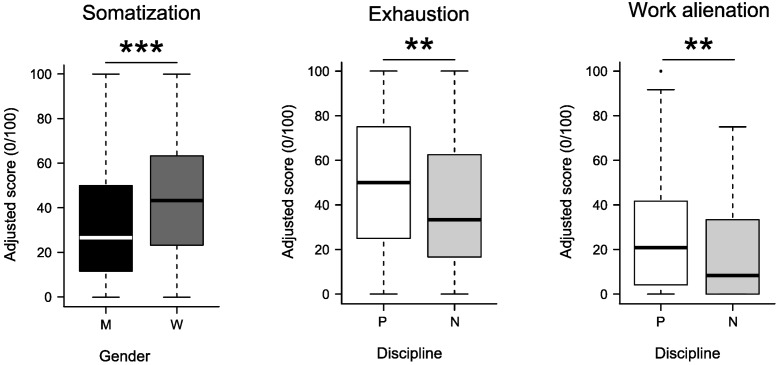
Differences confirmed from comparative analysis by gender, men (M) and women (W), and by discipline, physicians (P) and nurses (N), of the sub-scales of somatization, exhaustion, and work alienation; ^∗∗^*p <* 0.01; ^∗∗∗^*p <* 0.001.

Regardless the discipline (physician, nurse), results of the analysis of variance confirmed a significant main effect of gender (men, women) for this element [*F*_(1,349)_ = 12.67; *p* < 0.001], as is shown in **Figure 2**. No main effects were found when a two-way interaction of gender with discipline was performed. The summary of these results is reported in **Table 2**.

**Table 2:** Summary results of a Two-Way analysis of variance in a sample of 353 Latin American physicians and nurses.

Source of variation	*F*_(1.349)_			
	
	SCE	Somatization	Exhaustion	Work alienation
**Main effects**				
Gender (men vs. women)	2.42	12.67^∗∗∗^	0.22	0.41
Discipline (physician vs. nurse)	5.54^∗^	0.38	9.26^∗∗^	8.72^∗∗^
**Two-way interaction**				
Gender – Discipline	0.16	0.13	0.31	0.01


*SCE, Scale of Collateral Effects; ^∗^*p <* 0.05; ^∗∗^*p <* 0.01; ^∗∗∗^*p <* 0.001.*

On the other hand, correlation analysis confirmed the existence of an inverse association between somatization and age in the entire sample (*P* = –0.26; *p <* 0.001). Correlation analysis by groups of discipline (**Table 3**), show that in the group of physicians there are inverse associations between somatization and empathy (*P* = –0.17, *p* = 0.01), and between somatization and lifelong learning (*P* = –0.14; *p* = 0.04), but not between somatization and teamwork (*P* = –0.07; *p* = 0.32). This association was not evident in nurses neither between somatization and empathy (*P* = –0.14; *p* = 0.11), nor between teamwork and somatization (*P* = –0.05; *p* = 0.60).

**Table 3:** Spearman’s correlation analysis among collateral effects, empathy, teamwork, lifelong learning, and age in 353 physicians and nurses from four Latin American countries.

Variables	SCE	Somatization	Exhaustion	Work alienation
**Professionalism**				
**Empathy**				
Physicians	–0.16^∗^	–0.17^∗^	–0.12	–0.14^∗^
Nurses	–0.19^∗^	–0.14	–0.15	–0.23^∗∗^
**Teamwork**				
Physicians	–0.09	–0.07	–0.11	–0.02
Nurses	–0.07	–0.05	–0.03	–0.09
**Lifelong learning**				
Physicians	–0.18^∗∗^	–0.14^∗^	–0.13	–0.26^∗∗∗^
Nurses	+0.11	+0.16	+0.10	+0.02
**Age**				
Physicians	–0.22^∗∗∗^	–0.27^∗∗∗^	–0.16^∗^	–0.13^∗^
Nurses	–0.28^∗∗^	–0.28^∗∗∗^	–0.20^∗^	–0.23^∗∗^


*SCE, Scale of Collateral Effects; *^∗^p <* 0.05; ^∗∗^*p <* 0.01; ^∗∗∗^*p <* 0.001.*

### Exhaustion

Results of the variance analysis (**Table 2**), regardless of gender (men, women), showed significant main effects of the discipline (physician, nurse) for exhaustion [*F*_(1,349)_ = 9.26; *p* = 0.003]. Physicians reported higher exhaustion than nurses (**Figure 2**). No main effects were found with gender. No main effects for this element appeared when a two-way interaction was performed.

Correlation analysis confirmed an inverse association between exhaustion and age in the entire sample (*P* = –0.15; *p* = 0.006). This association appears in both groups of discipline separately (physicians and nurses), as is shown in **Table 3**. However, no association to exhaustion was confirmed in either group (physicians and nurses) when empathy, teamwork, or lifelong learning punctuations were assessed.

### Alienation

Regardless of the gender (men, women), results of the variance analysis showed significant main effects for the discipline [*F*_(1,349)_ = 8.72, *p* = 0.003]. No main effects were found with gender. No main effects were either found when a two-way interaction of gender by discipline was assessed (**Table 2**). Differences in punctuation between physicians and nurses are shown in **Figure 2**.

Age in physicians and nurses (*P* = –0.14, *p* = 0.01) was associated with less alienation. Empathy in physicians (*P* = –0.14, *p* = 0.04) and in nurses (*P* = –0.23; *p* = 0.009) was also associated with less alienation (**Table 3**). With regard to lifelong learning, it was associated to less alienation in physicians (*P* = –0.26; *p <* 0.001), but not in nurses (*P* = +0.02; *p* = 0.81). No association appeared from the correlation analysis between alienation and teamwork.

## Discussion

All instruments showed an adequate psychometric reliability with Cronbach’s alpha coefficients higher than 0.70. Those coefficients were slightly inferior to the originals for empathy (Hojat et al., 2002), lifelong learning (Hojat et al., 2009), and somatization (Blanch et al., 2010), but slightly superior to the originals for teamwork (Hojat et al., 2003), exhaustion (Blanch et al., 2010), work alienation (Blanch et al., 2010), and global perception of collateral effects (Blanch et al., 2010). Looking at **Table 1**, a potential ceiling effect was observed since the maximum possible punctuations of empathy, teamwork, and lifelong learning scales were reached by some respondents. Although those instruments have shown robust psychometric properties that have been demonstrated in different professional and cultural contexts, future studies can be performed to revised possible improvements in this direction.

No statistical differences were found for the scores of global perception of collateral effects, somatization, exhaustion, and alienation when they were compared by country. These findings confirm a similar perception of the three collateral effects measured in healthcare professionals from institutions of four different Latin American territories. These findings are also in consonance with those reported in a recent study with physicians from Colombia, Brazil, Chile, and Venezuela (Ochoa and Blanch, 2016), but not with those preliminary reported with healthcare professionals from Argentina and other Latin American countries (Grau et al., 2009), where Argentineans showed a higher perception of burnout. According to those authors (Grau et al., 2009), differences observed between Argentinean and other Latin American healthcare professionals may be associated to differences in socio-economical conditions in those countries, but also can be the consequence of a methodological limitation: an unbalanced geographical distribution of the study sample (65% of the entire sample was composed by the Argentinean group, while the other 35% included data collected in ten Latin American countries). In this sense, findings of this study offers updated and new evidence in support of a similar perception of occupational work in Latin American healthcare professionals despite potential socio-economical differences by territory.

A recently published review by Zacher and Schmitt (2016) remarks the positive role that age plays in occupational well-being. According to them, the lack of studies on work characteristics (i.e., social support or time pressure) as mediators of age-occupational well-being relationship is surprising. Our study provides evidence of a positive association between age and occupational well-being even when work characteristics are different, as it possibly occurs when physicians’ and nurses’ work characteristics are compared. Our findings are also in accordance with two models: “successful aging” and “role theory”. According to the “successful aging” model, successful aging at work involves a process during which workers maintain or improve favorable work outcomes, such as motivation, performance, and well-being with increasing age (Kooij et al., 2008; Zacher, 2015). On the other hand, the “role theory” model states that workers occupy multiple roles within and outside the work context, and that perception and perceived importance of these roles, and more specific tasks, expectations, and available resources within those roles, change over time and with age (Ashforth, 2001).

In physicians, punctuations observed for empathy and teamwork were slightly inferior than reported in previous studies with Latin American physicians-in-training (Delgado-Bolton et al., 2015; San Martín et al., 2016), but superior to those reported for lifelong learning (San Martín et al., 2016). Based on those differences, findings of this study are consistent with those reported in a previous cross-cultural study where the same specific elements of medical professionalism were compared between Spanish and Latin American physicians-in-training (San Martín et al., 2016). On the other hand, differences observed between physicians’ and nurses’ attitudes toward inter-professional collaboration in this study are consistent with those reported in previous studies with physicians and nurses from Mexican institutions (Hojat et al., 2001, 2003). Indeed, this study provides evidence of the prevalence of a “hierarchical” model in physician-nurse relationships not only in Mexican, as was first reported years ago (Hojat et al., 2001), but also in other Latin American institutions.

With regard to the role of the influence that the “hierarchical” work model plays in the relationship between occupational well-being and professionalism, the findings of this study confirm that not only nurses (Hakanen et al., 2014) but also physicians suffer higher risk of work distress when inter-professional relationships are hierarchical instead of complementary. Furthermore, important differences in the perception of exhaustion and work alienation between physicians and nurses in Latin American institutions reveal that physicians have a higher risk of suffering work distress than nurses. These findings, in consonance with those reported in a previous study on medical burnout in Latin American healthcare professionals (Grau et al., 2009), might be consequence of the bigger responsibilities and decisions that practitioners have to take in comparison with nurses. On the other hand, nurses under hierarchical working contexts are restricted to caring. In consequence, other abilities (especially those related to inter-professional collaboration and lifelong learning) could be often undervalued by their supervisors (Ogilvie et al., 2007) or by other nurses (Rooddehghan et al., 2015). This is also in accordance with what the JDC and JDCS models state about job demands, job control, and social support interactions, especially when they are coming from coworkers and supervisors (Karasek, 1979; Johnson and Hall, 1988; Karasek and Theorell, 1990; Pisanti et al., 2016).

In physicians, the more managerial and the greater the responsibility of their professional role may drive them to a greater development of abilities in research and information analysis, which they can apply to improve the diagnosis and treatment of their patients. It can explain their higher scores in lifelong learning in comparison with nurses. On the contrary, with the nurses’ collaborative role being limited to patient care, it may drive them to lose their motivation for developing learning abilities. In accordance with other authors (Tang et al., 2013), the findings of this study bring new evidence in support of how different roles and autonomy attached with these stereotypical ideals have made not only collaboration, but also learning, a stifling experience for many nurses. Under those circumstances, nurses who have strong commitments with the improvement of their professional competencies toward lifelong learning are the most vulnerable.

In nursing, as a healthcare profession that is oriented to the patients’ care, empathy has a central role (Mortier et al., 2016). Therefore, empathy is expected to be a characteristic found in all nurses, independently of their specific role (Hojat, 2016). This would explain why nurses had lesser distractions than the physicians concerning empathy, and why this ability is protective in prevention of work distress. A higher distraction concerning empathy in physicians may be caused by the nature of their clinical and medical roles and the professional duties associated to them.

However, regarding the differences in empathy scores between physicians and nurses, it is necessary to understand two important issues: the double nature of empathy and the healthcare contexts in Latin America. Empathy in the context of healthcare is defined as a cognitive (as opposed to emotional/affective) attribute that involves understanding the experiences and perspective of the patient, combined with the ability to communicate this understanding to the patients (Hojat and Gonnella, 2016). This distinction is very important since it has demonstrated that emotional empathy in nurses is positively correlated with emotional exhaustion (Williams, 1989), and with burnout (Vévodová et al., 2016). On the contrary, empathy as a cognitive attribute of caregivers has a positive role on prevention of burnout and work distress (Hojat, 2016). On the other hand, many Latin American public health institutions, healthcare professionals have to address daily social needs with scarce resources (Almeida, 2002; Cotlear et al., 2015). Under those circumstances, nurses are more exposed than physicians to patients’ daily concerns, complains, and frustrations (Grau et al., 2009). Under those circumstances, it is expected that nurses whom are emotionally intense with their patients build psychological barriers against any empathetic concern as the coping strategy (Hojat, 2016). Additionally, nurses, limited to patient care, may be less motivated to develop learning abilities that could help them in the improvement of their empathetic abilities with the patients. Both issues, one related to the working environment and the other to the coping strategy, can explain why in this study the entire group of nurses reported lower empathetic punctuations than physicians. Unfortunately, this coping strategy is counterproductive to nurses since findings of this study demonstrate that empathy plays a positive role in occupational well-being.

Finally, gender comparisons yield differences for somatization but not for exhaustion or work alienation. These findings are consistent with those of previous studies where somatization was compared between men and women in developing countries (Wool and Barsky, 1994; Piccinelli and Simon, 1997). Another study reported that the risk of somatization is slightly higher for women in comparison to men but it rapidly escalates when it is combined with low social class and high emotional distress (Ladwig et al., 2001). The findings of this study are in agreement with those previous observations, and underline important differences due to gender that are still prevalent in societies where hierarchical role models are dominant.

All these findings stress the importance that empathy, abilities toward inter-professional collaboration, and lifelong learning have in practitioners’ health and welfare, and the role that cultural behaviors play in the interaction between professionalism and occupational well-being.

## Ethics Statement

This study was carried out in accordance with the recommendations of tico de Investigacin Clinica de La Rioja with written informed consent from all subjects. All subjects gave written informed consent. The work was carried out in accordance with the Ethical principles for medical research involving human subjects of the Declaration of Helsinki, adopted by the World Medical Association. The protocol was approved by the tico de Investigacin Clinica de La Rioja (Ref. CEICLAR PI 199).

## Author Contributions

LV was in charge of the study’s overall design, coordination with the participating institutions, and drafting of the manuscript. MS-B and LV performed the statistical processing of data. All authors contributed to the presented work, participated during the interpretation process of the results, and approved the final manuscript.

## Conflict of Interest Statement

The authors declare that the research was conducted in the absence of any commercial or financial relationships that could be construed as a potential conflict of interest.
